# [Retracted] MicroRNA-519a inhibits the proliferation and promotes the apoptosis of ovarian cancer cells through targeting signal transducer and activator of transcription 3

**DOI:** 10.3892/etm.2025.13044

**Published:** 2025-12-09

**Authors:** Fei Tian, Ligang Jia, Zhaoping Chu, Hua Han, Yuan Zhang, Jianhui Cai

Exp Ther Med 15:1819–1824, 2018; DOI: 10.3892/etm.2017.5600

Following the publication of this paper, it was drawn to the Editor's attention by a concerned reader that areas of the flow cytometric (FCM) plots featured in Fig. 2C on p. 1822 were strikingly similar to FCM data which were ultimately published in a pair of other papers in different journals that were written by different authors at different research institutes, including an article that was submitted during the same week as this one to the journal *Biomedicine & Pharmacotherapy*.

Owing to the fact that the contentious data in the above article were found to be strikingly similar to data that were submitted for publication elsewhere at around the same time, the Editor of *Experimental and Therapeutic Medicine* has decided that this paper should be retracted from the Journal. The authors were asked for an explanation to account for these concerns, but the Editorial Office did not receive a reply. The Editor apologizes to the readership for any inconvenience caused.

